# Hollow Biomass Adsorbent Derived from Platanus Officinalis Grafted with Polydopamine-Mediated Polyethyleneimine for the Removal of Eriochrome Black T from Water

**DOI:** 10.3390/molecules29235730

**Published:** 2024-12-04

**Authors:** Zefeng Jiang, Tongyang Song, Bowen Huang, Chengqiang Qi, Zifu Peng, Tong Wang, Yuliang Li, Linjing Ye

**Affiliations:** 1 School of Water and Environment, Chang’an University, Xi’an 710054, China; 2023129042@chd.edu.cn (Z.J.); ty912634068@163.com (T.S.); 2022900286@chd.edu.cn (B.H.); 2022900612@chd.edu.cn (C.Q.); 18798260197@163.com (Z.P.); 18179000453@163.com (T.W.); yulianglee175@chd.edu.cn (Y.L.); 2 Key Laboratory of Subsurface Hydrology and Ecological Effect in Arid Region, Ministry of Education, Chang’an University, Xi’an 710054, China; 3 Key Laboratory of Eco-Hydrology and Water Security in Arid and Semi-Arid and Regions, Ministry of Water Resources, Chang’an University, Xi’an 710054, China

**Keywords:** Platanus officinalis, polydopamine, polyethyleneimine, graft copolymerization, adsorption

## Abstract

Platanus officinalis fibers (PFs) taking advantage of high-availability, eco-friendly and low-cost characteristics have attracted significant focus in the field of biomaterial application. Polyethyleneimine grafted with polydopamine on magnetic Platanus officinalis fibers (PEI-PDA@M-PFs) were prepared through a two-step process of mussel inspiration and the Michael addition reaction, which can work as an effective multifunctional biomass adsorbent for anionic dye with outstanding separation capacity and efficiency. The as-prepared PEI-PDA@M-PFs possess desirable hydrophilicity, magnetism and positive charge, along with abundant amino functional groups on the surface, facilitating efficient adsorption and the removal of Eriochrome Black T (EBT) dyes from water. In addition to the formation mechanism, the adsorption properties, including adsorption isotherms, kinetics, and the reusability of the absorbent, were studied intensively. The as-prepared PEI-PDA@M-PFs achieved a theoretical maximum adsorption capacity of 166.11 mg/g under optimal conditions (pH 7.0), with 10 mg of the adsorbent introduced into the EBT solution. The pseudo-second-order kinetic and Langmuir models were well matched with experimental data. Moreover, thermodynamic data ΔH > 0 revealed homogeneous chemical adsorption with a heat-absorption reaction. The adsorbent remained at high stability and recyclability even after five cycles of EBT adsorption processes. These above findings provide new insights into the adsorption processes and the development of biologic material for sustainable applications.

## 1. Introduction

Organic dyes in wastewater have obviously become a delicate issue in water resource protection and sustainable environment development [1,2,3]. These organic compounds are widely used in textiles, papermaking, cosmetics, leather, plastic, food and other industrial fields with complex and stable chemical structures, showing non-biodegradable, toxic and carcinogenic effects after prolonged exposure [4,5]. With the increasing dye wastewater crisis, various technologies have sought to solve dyes bearing effluents, such as flocculation [1,6], adsorption [7], electrochemistry [8,9], ion exchange [10], oxidation processes [11], biological treatments [12], etc. Among these technologies, the process of adsorption is progressing quickly and taking advantage of a wide range of raw materials, low costs, convenient treatment, and high efficiency [4,13,14,15].

In adsorption operations, natural biomass-based adsorbents have attracted widespread attention due to their feasibility and eco-friendliness in wastewater treatment [16,17,18]. Platanus officinalis fibers (PFs), derived from the seed of Platanus officinalis, predominantly consist of cellulose, hemicellulose and lignin arranged in a linear pattern. Platanus officinalis are widely planted in China for air pollution resistance, noise and dust reduction, as well as air purification [19]. However, when the Platanus officinalis seeds are ripe, each of the seeds, containing two to five million fibers, falls off and flies with the wind in April every year, which not only pollutes the environment but also causes people’s skin to itch, as well as causing allergic diseases, respiratory diseases, and other hazards [20,21,22]. These fibers possess diverse functional groups, such as carboxyl, hydroxyl and amino groups, enabling them to bond chemically with organic substances and heavy metal ions, including in wastewater [23,24,25,26,27]. The inherent hollow and tubular structure of PFs contributes to their substantial surface area and internal spatial arrangement, enhancing their adsorption capacity [28,29]. Nonetheless, compared to conventional counterparts like activated carbon or functionalized adsorbents, the pollutants’ adsorption efficiency in aquatic environments is not satisfactory considering the hydrophobic nature of PFs’ surface and the difficulties in their recycling efficiency.

Mussel-inspired surface modification is an effective way to enhance the wettability of biomass materials, with dopamine (DA) being a widely used modifier in this process [30,31,32]. It has been reported that DA can form adhesive polydopamine (PDA) films on both inorganic and organic material surfaces through self-polymerization in mildly alkaline conditions, resulting in improved hydrophilicity [33]. Furthermore, the chemical structure of PDA boasts various functional groups, including catechol, amino, and benzene ring, which can function as adsorption sites to augment its adsorption capabilities [34,35]. However, it should be noted that PDA, while widely employed for surface modification, exhibits limited performance in adsorbing anionic pollutants in water bodies, due to its structural instability in alkaline conditions and its inherent electronegativity [33]. Fortunately, PDA-modified surfaces can serve as a versatile platform for secondary modification. By introducing biomolecules with nucleophilic groups (e.g., R-NH_2_, R-SH), it is possible to further fine-tune the surface chemistry of biomass materials using reactions like the Michael addition or the Schiff base reaction [36,37]. This opens up opportunities to enhance the adsorption properties of PDA-modified materials for a broader range of pollutants in aquatic environments.

It has been shown that amino groups are easily protonated in aqueous solution and can adsorb anionic pollutants through electrostatic interactions, and are therefore considered to be outstanding functional groups for the removal of various anionic pollutants [38]. Polyethyleneimine (PEI), a water-soluble polymer enriched with amino functional groups along its molecular chain, exhibits electropositive properties in aqueous solutions. However, High water solubility and challenges in recycling impede its direct utilization for pollutant removal in water. Typically, PEI is chemically grafted onto or physically blended with other matrix materials for practical applications. For instance, Juang et al. [39] successfully grafted PEI onto a fiber membrane composed of chitosan and poly (vinyl alcohol) (PVA), achieving the selective removal of the anionic dye methyl orange, with an impressive maximum adsorption capacity of 70.8 mg·g^−1^.

In this paper, we used mussel inspiration and Michael addition to prepare adsorbents of Platanus officinalis fibers for the efficient removal of anionic pollutants from water with magnetic recovery. Specifically, cobalt ferrate with magnetic properties was loaded onto the surface of Platanus officinalis fibers using the hydrothermal method, while PDA and PEI were coated and grafted onto the surface of the material by a two-step impregnation method, respectively. The effects of several factors, containing solution pH, adsorbent dosage, initial concentration, and contact time, on the removal efficiencies of Eriochrome Black T (EBT) were investigated, followed by adsorption isotherm, kinetic, and thermodynamic studies carried out on the adsorption process. The corresponding EBT separation mechanism has been elaborated through a series of characterization analyses, such as FT-IR and XPS. Overall, the PEI-PDA@M-PFs synthesized through a facile modification displayed a novel performance of biomass adsorbents with excellent adsorption properties and ability to separate wastewater.

## 2. Experimental Section

### 2.1. Materials and Reagents

Raw Platanus officinalis fibers (R-PFs) were collected from Chang’an University’s Yanta Campus; sodium hydroxide (NaOH), Fe (NO_3_)_3_·9H_2_O and Co (NO_3_)_2_·6H_2_O were purchased from Tianjin Damao Chemical Reagent Technologies Co., Ltd. (Tianjin, China). Ethanol absolute (EtOH) was obtained from Tianjin Tianli Chemical Reagent Technologies Co., Ltd. (Tianjin, China). Dopamine hydrochloride (98 wt%) was purchased from Shandong Xiya Reagent Technologies Co., Ltd. (Linyi, China). Tris-(hydroxymethyl)-aminomethane (Tris–HCl) was purchased from Shanghai Macklin Biochemical Co., Ltd. (Shanghai, China). Polyethylene glycol (PEG8000), concentrated ammonia aqueous solution (NH_3_·H_2_O, 25%) and Eriochrome Black T (EBT) were purchased from Tianjin Yongsheng Fine Chemical Co., Ltd. (Tianjin, China). Polyethylene imine (70,000 Mw) was purchased from Shanghai Aladdin biochemical technology Co., Ltd. (Shanghai, China). All chemicals were of analytical grade and used directly without any further purification. The water used for experiments was deionized water. 

### 2.2. Synthesis of PEI-PDA@M-PFs Adsorbent

Before treatment, the raw Platanus officinalis fibers were macerated in 300 mL of 0.5% sodium hydroxide solution and stirred at 70 °C for 2 h. The preprocessed fibers were washed, filtered multiple times with deionized water and then dried in an oven at 60 °C for 24 h to obtain PFs.

The preparation of PDA@M-PFs was as follows: firstly, 200 mg of PFs was added into 40 mL of 5 mmol/L Co(NO_3_)_2_·6H_2_O and 10 mmol/L Fe(NO_3_)_3_·9H_2_O mixed solution, followed by adding 10 mL of ethanol solution containing 30 mg of PEG8000. After five minutes of ultrasonic dispersion, the pH of the mixture was adjusted to 10 and stirred for 2 h. The reaction solution was then transferred to a 100 mL PTFE-lined autoclave and reacted at 100 °C for 10 h to acquire magnetic PFs, namely M-PFs. Secondly, 80 mg of M-PFs was well dispersed in 20 mL of Tris-HCl buffer and then 40 mg of dopamine (DA) was dissolved in the mixture and stirred at 25 °C for 12 h. After that, the final product was washed with deionized water and anhydrous ethanol, and then dried in an oven for 12 h.

The preparation of PEI-PDA@M-PFs was as follows: the PEI solution was added dropwise into a glass beaker containing 50 mL of Tris-HCl (10 mmol/L, pH = 8.5) solution and 100 mg of the prepared PDA@M-PFs. The mixed solution continued to react for 5 h to complete the graft modification. After washing with deionized water and drying in an oven at 60 °C, the final PEI-PDA@M-PFs were obtained.

**Figure 1:**
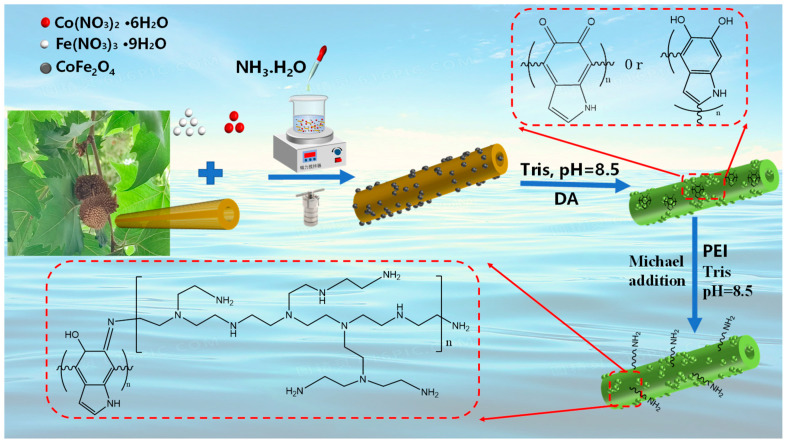
Schematic representations showing the fabrication procedures of the PEI-PDA@M-PFs.

The preparation of two-step processing is shown in Figure 1.

### 2.3. Characterizations of Materials

The structure and components of the as-prepared samples were characterized by a X-ray diffractometer (XRD, D/MAX-γ, Japan). The surface properties and morphological evaluation of the as-prepared samples were analyzed by a field emission scanning electron microscope (SEM, S-4800, Hitachi, Japan) and X-ray diffraction spectroscopy (XPS, Escalab Xi^+^, infrared, Thermo Scientific, USA). Thermogravimetric analysis of the as-synthesized samples was carried out in an oxygen atmosphere on a thermogravimetric analyzer (TG/DTG, PerkinElmer, USA). The magnetic properties and surface hydrophilicity of the materials were measured with a vibrating sample magnetometer (VSM, LakeShore, USA) and contact angle measurements (CA, JC2000D1, Powereach, China), respectively. The surface potential measurement of the materials was recorded on a Zeta Potentiometer (Zetasixer, ZEN3700, Malvern, UK). Dye adsorption experiments were carried out on a Shimadzu UV-1200 spectrophotometer. Infrared spectroscopy was carried out on a Bruker Optics ALPHA-E infrared spectrometer.

### 2.4. Batch Adsorption Experiments

The procedure of batch adsorption study was performed to evaluate adsorption behaviors, the effects of pH value (2–10), adsorption time (0–1500 min), adsorbent dosage (50 mg/L, 75 mg/L, 100 mg/L), the initial dye concentration (20–150 mg/L) and solution temperature (293 K, 303 K, 313 K, 323 K, and 333 K) upon removing EBT. Typically, the as-prepared sample was added into a conical flask containing 25 mL of EBT solution with a certain concentration, followed by being shaken at 200 rpm in a water bath shaker with a fixed temperature. After that, the adsorbent was separated from the solution with a magnet and the residual EBT concentration in the solution was analyzed through a UV-Vis spectrophotometer with a wavelength of 546 nm. The amount of EBT adsorption and adsorption efficiency were calculated based on Equations (1)–(3).

(1) $$q_{t}=\frac{\left(C_{0}-C_{t}\right)\cdot V}{m}$$

(2) $$q_{e}=\frac{\left(C_{0}-C_{e}\right)\cdot V}{m}$$

(3) $$R\%=\frac{\left(C_{0}-C_{e}\right)}{C_{0}}\times 100\%$$

where *q_t_* and *q_e_* are the amount of EBT adsorption corresponding to a adsorption time (*t*) and reaching adsorption equilibrium (mg/g), respectively. *R* stands for adsorption efficiency, %. *C*_0_, *C_t_* and *C_e_* are the concentrations at the initial time, at *t* and at equilibrium of the dye (mg/L), respectively. *V* (L) is the volume of the solution and *m* (g) is the mass of adsorption materials.

### 2.5. Reusability Experiments

The reusability of the adsorbent was evaluated after adsorption with an initial concentration of 50 mg/L EBT solution, then 10 mg of the adsorbent was separated by a magnet and regenerated in a 0.1 mol/L NaOH solution with continuous stirring for 24 h. The regenerated adsorbent was reintroduced into the EBT solution again with a concentration of 50 mg/L for another EBT adsorption process. The above steps were repeated five times to determine the recycling performance.

## 3. Results and Discussion

### 3.1. Chemical Structure and Morphology Characterization of PEI-PDA@M-PFs

**Figure 2:**
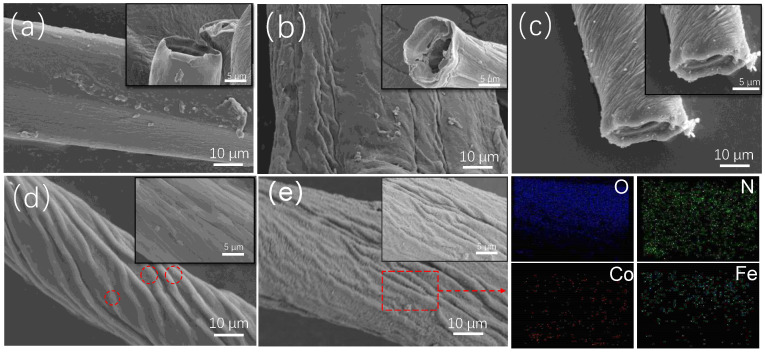
SEM images showing the morphology of R-PFs (**a**), PFs (**b**), M-PFs (**c**), PDA@M-PFs (**d**) with enlarged area in red cicles, PEI-PDA@M-PFs (**e**) and elemental mapping from red box.

The characteristics of the surface morphology of PEI-PDA@M-PF composites were observed by SEM during the preparation processes. In Figure 2a,b, the surface of PFs exhibits noticeable traces of roughening post-alkali treatment, showing that the groove stripes along the fiber axis increased along with the hollow structure. When the M-PFs were prepared, CoFe_2_O_4_ particles deposited on the surface and holes of the PFs could be found in Figure 2c. Figure 2d illustrates a membrane-like structure covering CoFe_2_O_4_ particles with pronounced grooves in on the surface of M-PFs after the M-PFs were immersed in the DA solution, indicating a successful PDA coating on the M-PFs. The CoFe_2_O_4_ particles covered by the membrane can be seen from the magnified areas circled in red. After adding PEI grafted with PDA, as presented in Figure 2e, a layer of fluffy material assembled on the grooves and ridges, leading to the surface of PEI-PDA@M-PFs denser than that of PDA@M-PFs, implying that PEI was successfully loaded onto the surface of PDA@M-PFs. Additionally, elemental distribution in elemental mapping confirmed the presence of oxygen, nitrogen, iron and cobalt.

**Figure 3:**
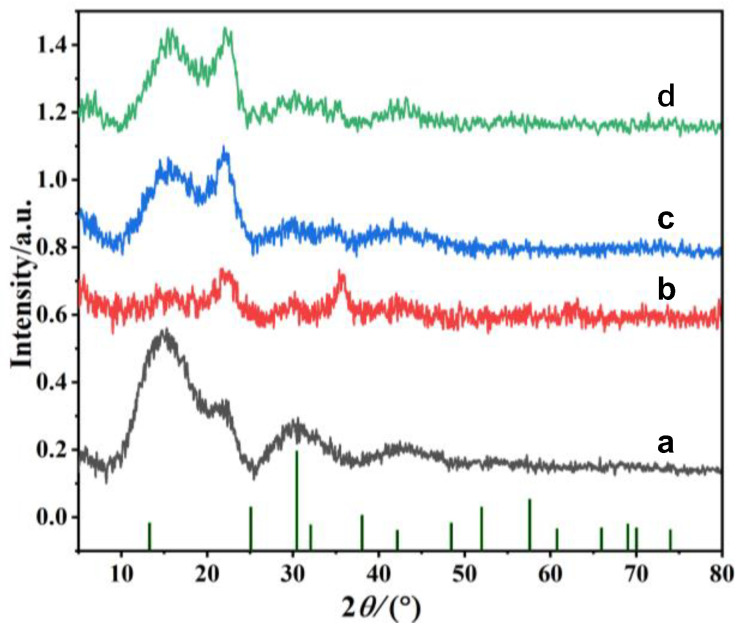
XRD patterns of PFs (a), M-PFs (b), PDA@M-PFs (c) and PEI-PDA@M-PFs (d).

The crystal structure of PFs, M-PFs, PDA@M-PFs and PEI-PDA@M-PFs was characterized, and the results are shown in Figure 3. The PFs demonstrate a predominantly amorphous structure with low crystallinity, displaying prominent diffraction peaks at 2θ of 13.46° and 22.12°, which correspond to (110) and (002) crystal planes, indicating a cellulose type I crystalline form [40]. The XRD pattern of M-PFs displayed the typical diffraction peaks of cubic structures with 2θ of 35.47°, 42.06°, 56.93° and 62.04°, attributed to (311), (400), (511) and (440) planes of CoFe_2_O_4_ (JCPDS:22-1086). The diffraction peaks of CoFe_2_O_4_ were weakened, obviously owing to the encapsulation of PDA in PDA@M-PFs, while distinct peaks emerged at 15.55° and 21.23°, corresponding to the amorphous structure of PDA [41]. When PEI is grafted onto the surface of the material, the XRD spectrum remains substantially unchanged, because PEI only regulates the structural properties of the material, without generating any crystalline substances [38]. The above findings confirm the presence of CoFe_2_O_4_ particles on the surface of PFs and that the PDA worked as an anchor connecting PEI onto the surface of PDA@M-PFs.

**Figure 4:**
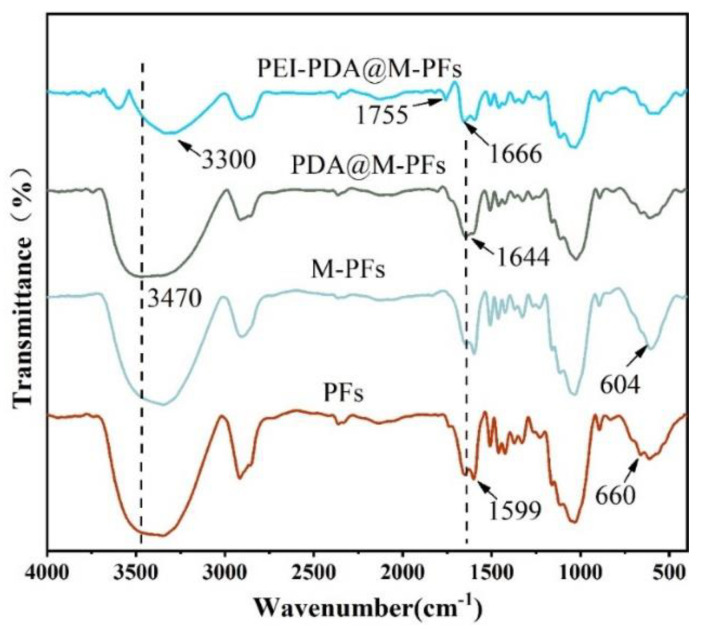
FT-IR spectra of PFs, M-PFs, PDA@M-PFs and PEI-PDA@M-PFs.

In order to ascertain the successful synthesis of PEI-PDA@M-PFs, FT-IR spectra were employed to analyze the chemical structure shown in Figure 4. The characteristic peaks of PFs at 3347 cm^−1^, 1644 cm^−1^, 1599 cm^−1^, 660 cm^−1^, 1030 cm^−1^ and 612 cm^−1^ correspond to the vibrational absorption peaks of O-H, N-H, C=O, C=C, C-N and C-O, respectively [20,42]. Upon loading the PFs’ surface with CoFe_2_O_4_ particles, there was a noticeable increase in peak intensities at 604 cm^−1^ and 430 cm^−1^, attributable to the vibrational absorption peaks of Fe-O and Co-O bonds in CoFe_2_O_4_ [43]. Coating the magnetic Platanus officinalis fibers with polydopamine led to a peak at 1644 cm^−1^, corresponding to the C=O of polydopamine. The telescopic vibrational peak of the phenolic hydroxyl group Ph-OH appeared at 1268 cm^−1^, which is inherent in dopamine’s structure [44]. Furthermore, the polydopamine coating caused the weakening of all vibrational peaks of M-PFs, due to the shielding effect of the polydopamine tinting layer [34,45]. Upon grafting PEI onto the material’s surface, the O-H bond peak at 3470 cm^−1^ appeared to weaken, with a noticeable blue shift. The Michael base reaction between PDA and PEI enabled the C=O bond of PDA to weaken at 1644 cm^−1^, and stretching vibrational peaks of the C=N bond to appear at 1657 cm^−1^, indicating successful PEI grafting onto the material’s surface [39,46,47]. Additionally, the emerging peak at 1755 cm^−1^, attributed to the stretching vibration of -NH_2_ and the antisymmetric peak of NH^3+^, further confirms that PEI modification introduces additional amino and amino groups onto the PDA@M-PF surface [48].

**Figure 5:**
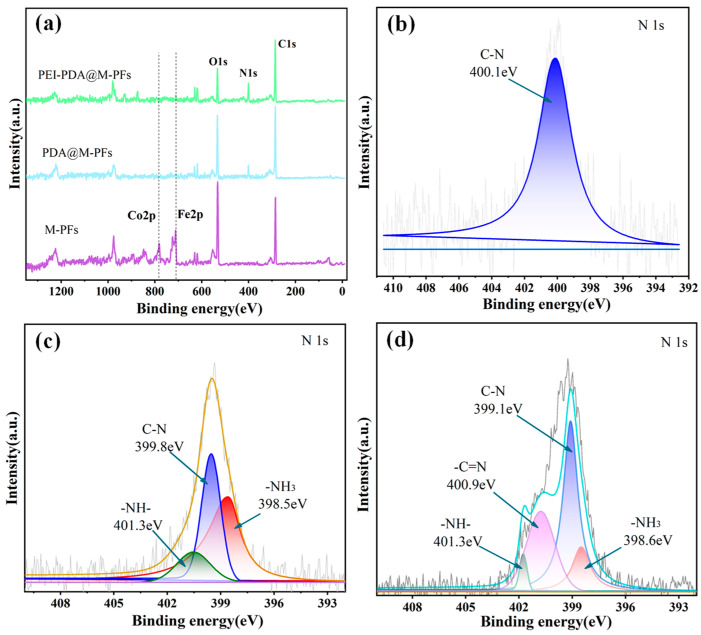
XPS wide-scan spectra (**a**) and N1s spectra of M-PFs (**b**), PDA@M-PFs (**c**) and PEI-PDA@M-PFs (**d**).

XPS analysis was used to discern the chemical composition and electronic states of material elements, and the results are presented in Figure 5a and Table 1. The peaks at 285.4 eV, 531.5 eV and 401.9 eV in the XPS full spectrogram signify C1s, O1s and N1s, respectively [36]. In the spectrograms of M-PFs, Fe2p and Co2p peak at 711.2 eV and 781.7 eV, with atomic occupancies of 3.83% and 0.99%, confirmed that the CoFe_2_O_4_ particle was synthesized and incorporated onto the PFs’ material surface successfully. Following PDA and PEI surface modification, the peak intensities of C1s and O1s diminished, while those of N1s increased. The percentage of surface N element surged from 1.09% to 11.36%, attributed to enhanced amino groups from PDA and PEI modification [49,50]. Simultaneously, the peak intensities of Fe and Co gradually weakened during surface modification, with elemental content decreasing to 0.33% and 0.24%, respectively, which was probably due to the shielding effect of PDA and PEI membranes [40].

Advantage software was utilized to peak split the fine spectral curves of N1s in the materials depicted in Figure 5b–d. The N1s curves of M-PFs exhibited a single peak at 399.9 eV, signifying the N-C bond [51]. Following PDA coating on the PDA@M-PFs, the N1s spectrum displayed three peaks at 400.6 eV, 399.5 eV and 398.6 eV, corresponding to -NH-, N-C and -NH_2_, respectively. Subsequent PEI grafting on the material surface of PEI-PDA@M-PFs resulted in an N1s spectrum with four peaks at 401.3 eV, 400.7 eV, 399.1 eV and 398.5 eV, representing -N=C, -NH-, N-C and -NH_2_, respectively. Comparing the peak strengths of -NH- and N-C in PDA@M-PFs, the spectral curves of PEI-PDA@M-PFs provided strong evidence for the grafting of PEI onto the PDA@M-PF surface [52].

**Figure 6:**
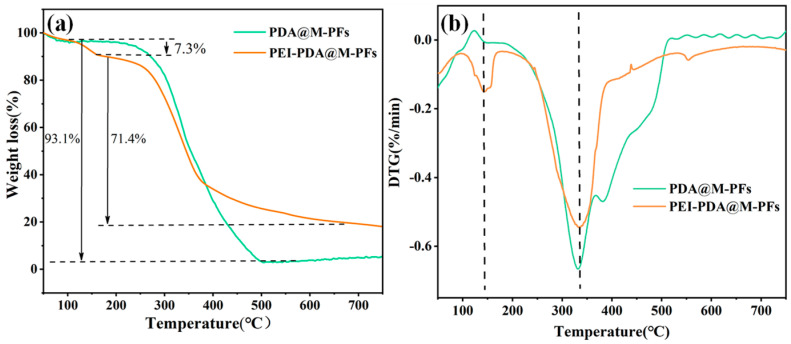
TGA (**a**) and DTG (**b**) curves of PDA@M-PFs before and after grafting of PEI.

The thermal decomposition of PDA@M-PF and PEI-PDA@M-PF materials was investigated through thermogravimetric analysis to assess thermal stability, and the TG/DTG analysis results are displayed in Figure 6. Thermogravimetric curves illustrated the mass changes before and after grafting in Figure 6a. It can be seen that both PDA@M-PFs and PEI-PDA@M-PFs had a slight mass loss in the temperature range of 50 to 100 °C because of the water evaporation [53]. The PDA@M-PF sample shows a main weight loss of 93.1% corresponding to the thermal decomposition of cellulose, hemicellulose and PDA surface coating [54]. While the thermal degradation temperature in TGA of the PEI-PDA@M-PF sample shows a two-step thermal loss process, one occured at about 130–180 °C with a weight loss of about 7.3%, due to thermal PEG decomposition, and the other occured at about 180–650 °C with a mass loss of 71.4%, revealing the thermal decomposition of both the PDA and PFs’ skeletons. The residual ash mass after grafting increased to 19.2% compared to pre-grafting. The DTG curves shown in Figure 6b show that the grafted material had a notable weight loss rate peak at 145 °C, which suggests a chemical reaction between PDA and PEI on the material’s surface, corresponding to the thermal decomposition of the polymer PEI [55]. In summary, the reduced weight loss in the grafted material indicates enhanced thermal stability.

**Figure 7:**
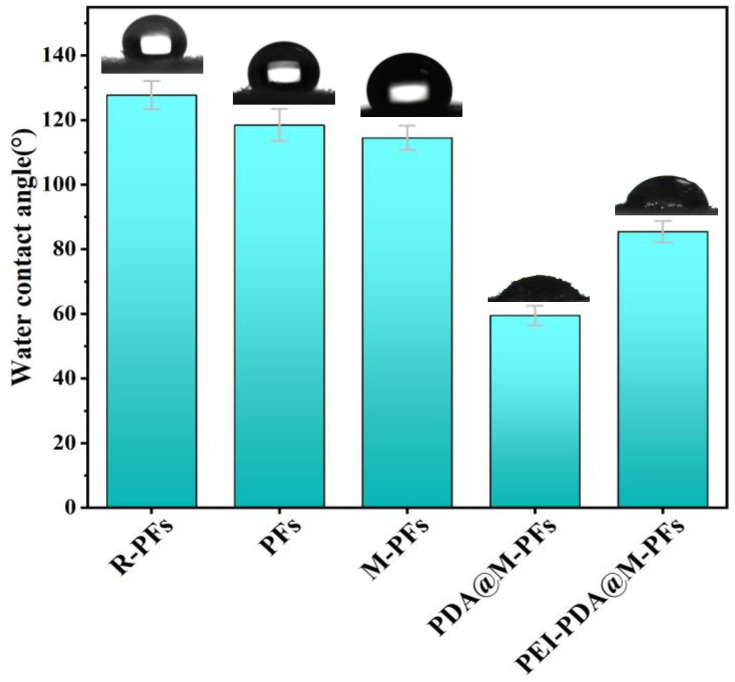
Water contact angle images of different materials.

This study tested the contact angles to evaluate the hydrophilicity of the samples as illustrated in Figure 7. The raw Platanus officinalis fruit fibers exhibited hydrophobicity with a water contact angle of 127.7°. The contact angle of R-PFs decreased slightly to 118.5° after alkali pretreatment, while the contact angle of M-PFs decreased to 114.5° after loading cobalt ferrate particles. The PDA@M-PFs are hydrophilic, as the polydopamine coating significantly improved the hydrophilicity, reducing the contact angle to 59.5°. This enhancement can be attributed to PDA’s abundant hydroxyl and amine groups, imparting hydrophilicity to the material [56], whereas the water contact angle of PEI-PDA@M-PFs increased to 85.5° with PEI grafting, possibly due to the weaker negativity charge of nitrogen atoms in the amino group compared to oxygen atoms in the hydroxyl group [46,57]. Overall, the existence of hydroxyl and amine groups in PDA combined with PEI finally increases the surface charge density and the hydrophilicity of PDA@M-PF surfaces. These alterations in water wettability on the material’s surface provide the foundation for waterborne dye adsorption [58].

**Figure 8:**
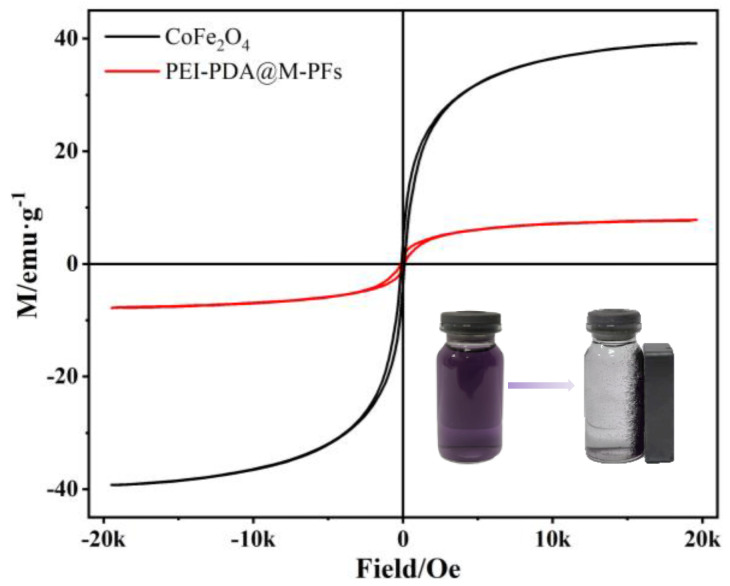
VSM curves of PEI-PDA@M-PFs and CoFe_2_O_4_ (the illustration shows the materials before and after magnetic separation at the end of adsorption).

The ferromagnetic behaviors of PEI-PDA@M-PFs and CoFe_2_O_4_ are shown in Figure 8. These hysteresis curves exhibit clear S-shaped patterns, indicating ferromagnetic properties, with saturation magnetization of 7.64 emu/g and 37.68 emu/g for PEI-PDA@M-PFs and CoFe_2_O_4_, respectively. This reduction is probably due to the non-magnetic nature of Platanus officinalis fibers and PDA incorporated into the structure. This new type of adsorbent shows powerful magnetic responsivity compared with other biomass-based absorbents, even though the PEI and PDA are incorporated into the structure. The schematic diagram in the lower-right corner illustrates that the adsorbent could be recycled conveniently from the EBT dye solution using an external magnet. This visual representation underscores the efficient magnetic separation of the adsorbent from water, simplifying the adsorbent’s recycling process.

### 3.2. Adsorption Properties and Kinetics of PEI-PDA@M-PFs

**Figure 9:**
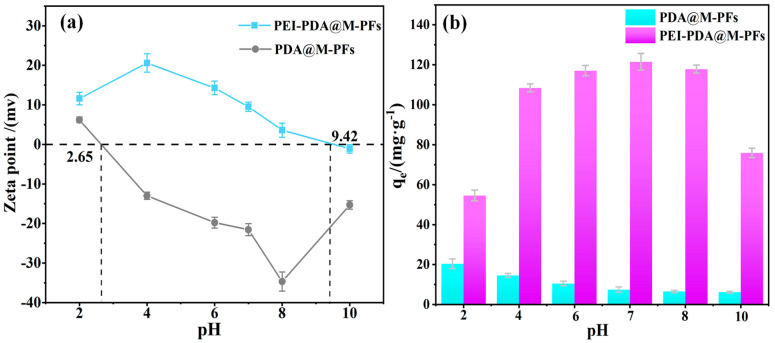
(**a**) Zeta point of PDA@M-PFs before and after grafting of PEI, (**b**) the effect of pH on adsorption capacity.

The effects of solution pH on EBT dye removal as well as on the adsorbent’s surface charge feature were investigated. As shown in Figure 9a, it is evident that PEI grafting significantly enhances the adsorbent’s positive-charge feature. The enhancement of point of zero charge (pH_pzc_) rises from 2.65 to 9.42, owing to the PEI’s stronger protonation effect compared to PDA [59]. Moreover, the material’s positive-charge feature remains relatively stable beyond pH 8, possibly due to the improved stability conferred by PEI grafting. The increased positive-charge feature and alkali stability provide a more suitable adsorption environment for the anionic dyes in water environment [60].

Adsorption measurements were conducted on the materials before and after PEI grafting, as depicted in Figure 9b. The material’s affinity for EBT notably increased post-PEI grafting. Furthermore, the adsorption capacity was gradually enhanced with increasing the solution pH from 2 to 7. At pH 7, the maximum adsorption capacity reached 121.21 mg/g. This enhancement could be attributed to the adsorbent’s positive charge surface and the electrostatic interactions between the anionic dye molecules [61]. However, contrary to expectations, the material exhibited inferior adsorption capacity at pH 4 at maximal Zeta potential, which suggests the presence of interaction forces beyond electrostatic interactions (e.g., physical adsorption, hydrogen bonding interactions, π-π interactions and surface complexation) [62].

**Figure 10:**
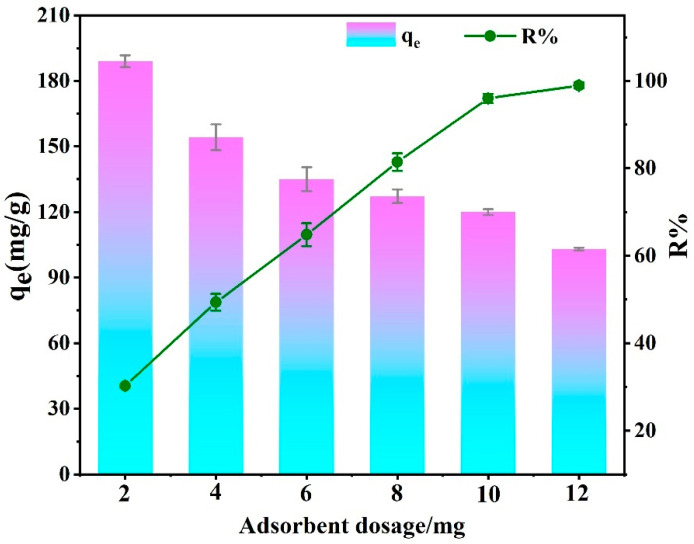
Effect of different PEI-PDA@M-PFs dosages on adsorption capacity.

Figure 10 illustrates EBT adsorption capacity and removal rate using different masses (2 mg, 4 mg, 6 mg, 8 mg, 10 mg and 12 mg) of the adsorbent with the dosage increased. The removal rate of EBT rose from 29.94% to 98.54%, while the adsorption capacity of the adsorbent for EBT gradually decreased with the adsorbent dosage increased The surplus of adsorbent provided more available adsorptive sites for dye molecule removal but also led to agglomeration [63]. The removal rate reached equilibrium after exceeding 10 mg, so 10 mg was selected as the fixed adsorbent dose, which was used in the following adsorption tests.

**Figure 11:**
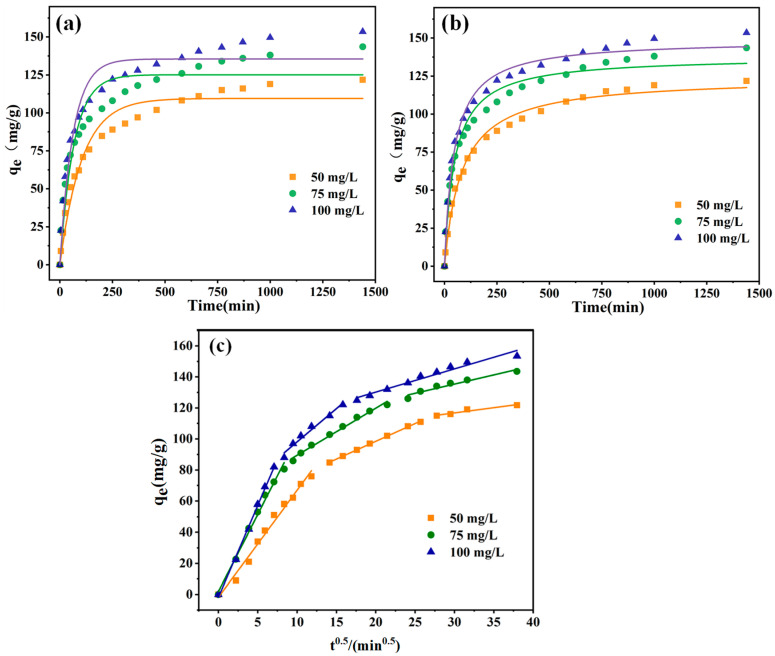
Adsorption kinetics of different concentrations of EBT on the surface of PEI-PDA@M-PFs; (**a**) pseudo-first-order model, (**b**) pseudo-second-order model and (**c**) in-particle diffusion model.

Various EBT solution concentrations were evaluated with 10 mg of PEI-PDA@M-PFs to study the adsorption kinetics, and the data were analyzed using the pseudo-first-order model (Equation (4)) and pseudo-second-order model (Equation (5)). The outcomes are presented in Figure 11 and Table 2.

The kinetic model based on the pseudo-first-order model is as follows:

(4) $$q_{t}=q_{e}\left(1-e^{-tK_{1}}\right)$$

The kinetic model based on the pseudo-second-order model is as follows:

(5) $$q_{t}=\frac{K_{2}\cdot q_{e}^{2}\cdot t}{1+K_{2}\cdot q_{e}\cdot t}$$

In these formulas, *q_t_* is the adsorption amount of dye at time *t*, mg·g^−1^. *q_e_* is the equilibrium adsorption amount, mg·g^−1^. *t* is the adsorption time, and min. *K*_1_ and *K*_2_ are the reaction rate constants.

As shown in Figure 11a,b, the adsorbent exhibited a fast adsorption rate during the initial 150 min, notably accelerated by higher pollutant concentrations because of the substantial concentration disparity between the dyes in the solution and those on the fibers in the early stages of adsorption, facilitating the rapid diffusion of dye molecules [64,65]. As the time progressed from 150 to 1000 min, the adsorption amount gradually approached equilibrium, with the maximum adsorption capacities for the adsorbent at different dye concentrations being 117.32 mg/g, 138.45 mg/g and 149.10 mg/g, respectively.

From Table 2, the fitting *R*^2^ values for the pseudo-second-order model (0.9844–0.9964) are better than those of the pseudo-first-order model (0.9134–0.9554). It is evident that the non-linear regression curves of adsorption efficiency aligned more closely with the pseudo-second-order kinetic model. Furthermore, the theoretical adsorption quantities derived from the pseudo-second-order model matched the experimental results well. This alignment underscored the predominant significance of chemical adsorption in the adsorption of EBT dye by the adsorbent. However, under different concentrations, the high *R*^2^ values of the pseudo-first-order model exceed 0.9, indicating the possibility of physical adsorption, such as the van der Waals force interaction, caused by surface magnetic particles [66].

In order to further explore the kinetic model as well as the adsorption mechanism of the adsorption process, the adsorption data were fitted to the in-particle diffusion model as well. The results are shown in Figure 11c. The EBT adsorption process is mainly composed of three stages, including surface diffusion, in-particle diffusion and adsorption equilibrium. The first step is the transfer of EBT molecules from the solution to the adsorbent surface. In the second step, a large number of EBT molecules occupied the adsorption site leads to a decrease in adsorption rate. The third step is that the adsorption gradually reaches an equilibrium, and the adsorption amount tends to reach the maximum [67]. Considering the parameter results in Table 3, *Kd*_1_ > *Kd*_2_ > *Kd*_3_ indicates that the rate of adsorption process is related to the adsorption equilibrium of in-particle diffusion, which also confirms that the physical adsorption of internal diffusion exists in the adsorption process.

**Figure 12:**
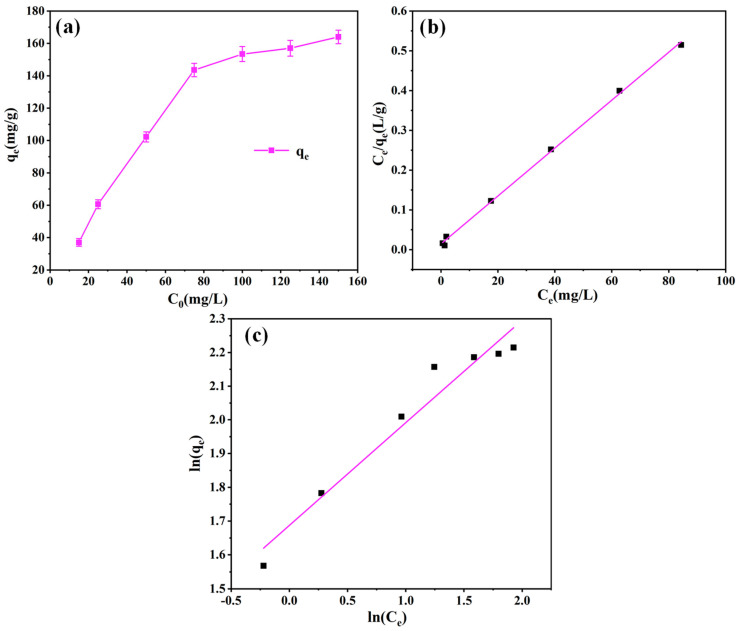
(**a**) Effects of the concentration of BET on adsorption properties of PEI-PDA@M-PFs and linear isotherm plots for the adsorption of EBT on PEI-PDA@M-PFs; (**b**) Langmuir, (**c**) Freundlich.

The effects of the initial dye concentration of ST were also considered. The adsorption of PEI-PDA@M-PFs with different concentrations of EBT was measured at temperature T = 298 K. The Langmuir isothermal model (Equation (6)) and Freundlich isothermal model (Equation (7)) were used to fit the data, and the results are shown in Figure 12 and Table 4.

Langmuir isotherm model:

(6) $$\frac{C_{e}}{q_{e}}=\frac{1}{q_{m}b}+\frac{C_{e}}{q_{m}}$$

Freundlich isotherm model:

(7) $$logq_{e}=logK_{F}+\frac{1}{n}logC_{e}$$

In these formulas, *q_m_* is the maximum adsorption capacity, mg·g^−1^. *C_e_* is the equilibrium–mass concentration and mg·L^−1^. *b*, *K_F_*, and *n* are adsorption-related constants.

A non-linear correlation between the adsorption performance of the adsorbent and EBT concentration is illustrated in Figure 12a. At first, the swift initial capacity escalation resulted from an augmented adsorption driving force, linked to a rise in pollutant concentration. Subsequently, the adsorption equilibrium reached over 100 mg/L. The whole process suggested a finite maximum adsorption capacity for the adsorbent, which was intricately linked to pollutant concentration [68,69].

The isothermal adsorption data were analyzed using the equilibrium adsorption isothermal model to elucidate the adsorption mechanism of the adsorbent on EBT. The outcomes are presented in both Figure 12b,c and Table 4. The results indicate a uniform distribution of the adsorption data on both sides of the Langmuir curve. Notably, the values of the correlation for the Langmuir model (*R*^2^ = 0.9984) surpass that of the Freundlich model (*R^2^* = 0.9456). This discrepancy underscores the dominance of monolayer adsorption in the adsorbent’s interaction with EBT. Moreover, employing the Langmuir model allowed for the computation of the maximum theoretical adsorption capacity of EBT dye by the adsorbent, yielding *q*_*e*,*max*_ = 166.11 mg/g.

**Figure 13:**
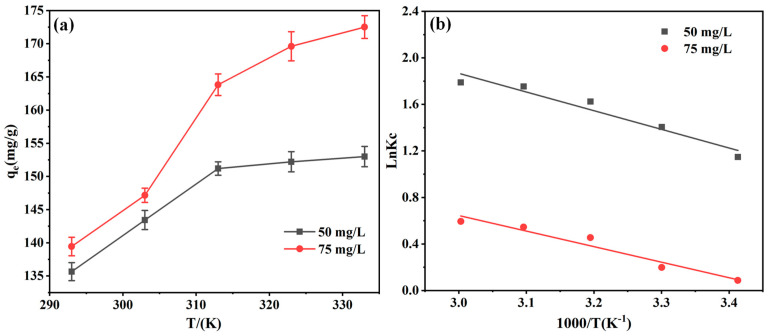
(**a**) Effect of temperature on the adsorption properties of PEI-PDA@M-PFs and (**b**) thermodynamic model.

The temperature dependence on the adsorption efficiency was investigated and the results are depicted in Figure 13. In Figure 13a, the uptrend in EBT adsorption by the adsorbent with rising temperature suggests a favorable temperature-dependent reaction. The thermodynamic model was applied to compute adsorption data, and the results are shown in Figure 13b and Table 5. Notably, ΔG remains negative from 293 K to 333 K, and the ΔG value turns increasingly negative with higher temperatures, which indicates that the EBT adsorbed on the PEI-PDA@M-PFs was spontaneous and appears to be a heat-absorbing nature of the absorbing process [62]. Moreover, the ΔH value is positive, which further confirms the heat-absorbing process, coordinated with dominance of chemical adsorption in the reaction. Higher temperatures facilitate the reaction progression. The positive ΔS suggests an increase in system disorder during the adsorption process, adding depth to our understanding.

**Figure 14:**
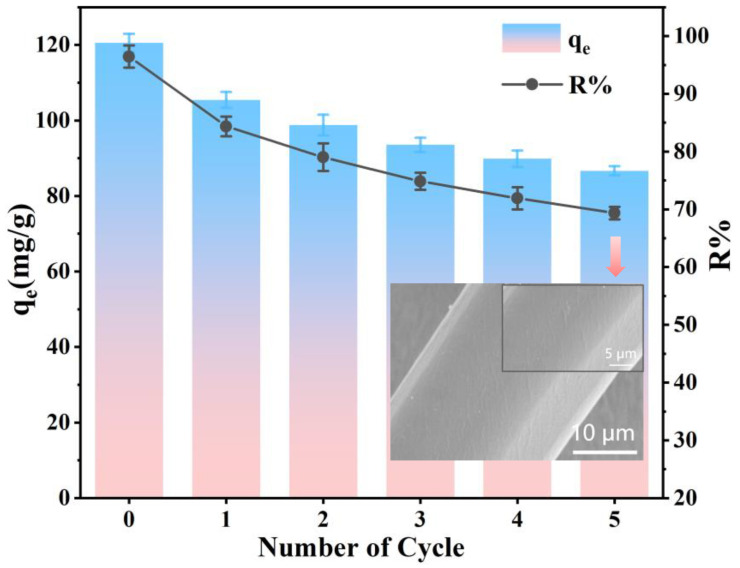
Reusability of PEI-PDA@M-PFs toward EBT (the illustration shows SEM images of PEI-PDA@M-PFs after five cycles).

In wastewater treatment, an ideal adsorbent not only takes into consideration the removal efficiency but also the reasonableness and recyclability [70]. To assess the regeneration capacity and stability of the PEI-PDA@M-PF adsorbent, five adsorption–desorption experiments were conducted, with the results depicted in the accompanying Figure 14. Following five cycles of adsorption and desorption, a marginal reduction in adsorption performance was observed. The inset SEM figure displays the smooth surface of the adsorbent after five cycles, due to the organic layer on the surface of the adsorbent partially degrading in the alkaline solution and falling off under mechanical operation, the change in the surface morphology of PEI-PDA@M-PFs is inevitable [26,27]. This reduction in rough gullies may result in a slight decrease in adsorption performance. However, the adsorption capacity remained as high as 86.69 mg/g, with an impressive EBT dye removal rate of 69.35%.

Compared with the adsorption of EBT in published research findings, the maximum adsorption capacity of Ni/Al-layered double hydroxides is 37.71 mg/g [71]. A chemically modified steel-powder-based adsorbent presents a maximum adsorption capacity of 119.02 mg/g [72], and a triazine resin adsorbent has a maximum adsorption capacity of 198.3 mg/g [73]. The biomass adsorbent prepared in our study demonstrates a competitive adsorption performance, with a maximum adsorption capacity of 121.21 mg/g. Besides the higher adsorption capacity, our material also exhibits better reusability, which highlights its potential for practical applications.

### 3.3. Adsorption Mechanisms

**Figure 15:**
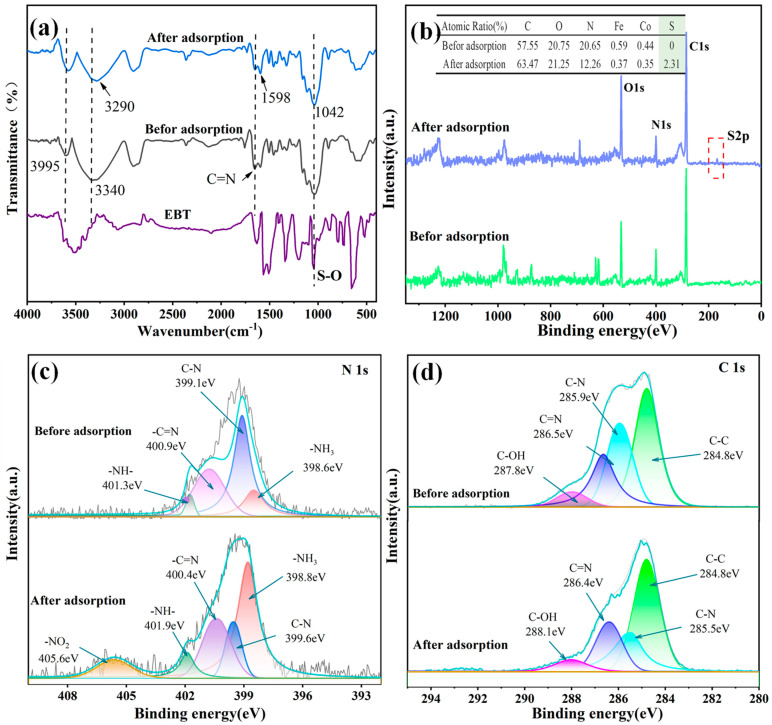
Characterization results of PEI-PDA@M-PFs before and after EBT adsorption: (**a**) FT-IR spectra, (**b**) XPS wide-scan spectra, (**c**) N1s spectra and (**d**) C1s spectra.

Considering the preceding findings from the preceding studies of the kinetics, isotherms, and thermodynamics of the adsorption process on the PEI-PDA@M-PFs, chemical adsorption plays a dominant role, augmented by electrostatic and van der Waals’ force interactions. For a better understanding of the intricacies of EBT dye adsorption on the PEI-PDA@M-PFs, we conducted a post-saturation characterization of the adsorbent using IR and XPS, with the results presented in Figure 15.

The infrared spectra shown in Figure 15a depict the pure EBT and the material before and after EBT adsorption, in which certain peaks shift and change. Both O-H and N-H group peaks exhibited noticeable blue shifts after EBT adsorption. Specifically, the N-H group’s broad peak shifted from 3340 cm^−1^ to 3290 cm^−1^, indicating facile protonation, rendering the material’s surface positively charged. Simultaneously, the -SO^3−^ of ionized sulfonic acid group in EBT dye solution electrostatically interacted with the positively charged protonated amine group [74]. The heightened peak intensity at 1042 cm^−1^, observed both pre- and post-adsorption, was attributed to EBT adsorption and the stretched vibration of the S-O bond in the EBT dye structure, which emphasizes the successful adsorption of EBT dye molecules by the material [75,76]. Furthermore, the sharp O-H peak at 3995 cm^−1^ shifted to 3575 cm^−1^, indicating hydrogen bonding interactions, with the N-containing group of EBT dye acting as a hydrogen acceptor [77]. Because EBT molecules existed on the surface of the adsorbed material, the C=N bond peak at 1657 cm^−1^ weakened due to π-π interactions between surface C=N bonds and dye molecules. Simultaneously, the vibrational peak of phenyl at 1598 cm^−1^ intensified, reflecting the numerous benzene rings in the adsorbed EBT molecules.

To delve deeper into the adsorption mechanism, we employed X-ray Photoelectron Spectroscopy (XPS) to examine the surface elemental intensities and the C1s and N1s fine spectra of the material pre- and post-EBT adsorption (Figure 15b). The full spectrum and the elemental quantitative analysis table revealed considerable changes in the diffraction peak intensities of C1s, N1s and O1s on the material’s surface, followed by EBT adsorption. Particularly, the C1s peak intensified while the N1s peak diminished, which is aligns with EBT’s molecular structure of rich in carbon elements as well as the affinity of O- and N-containing groups in EBT molecules. The material and O-containing groups have a preferential interaction than N-containing groups in the adsorption process of EBT. Additionally, the spectrum of the adsorbed material exhibits a distinct S2p peak at 168 eV, owing to the sulfonic acid group in EBT’s structure. This finding aligns seamlessly with the FT-IR analysis results, providing further support for the comprehensive understanding of the adsorption process [75].

The high-resolution fine spectra of N1s and C1s of PEI-PDA@M-PFs before and after EBT adsorption are presented in Figure 15c,d. The XPS spectra post-EBT adsorption revealed a distinct de-convolution peak of -NO_2_ at a binding energy of 405.6 eV, confirming the presence of -NO_2_ in the EBT molecule presented on the material’s surface. Furthermore, there are notable shifts in the peaks of -NH_3_ and -NH- to 398.8 eV and 401.9 eV, shown on the pre- and post-adsorption spectra, respectively. The increased peak area of -NH_3_ with elevated concentrations provided additional evidence for strong electrostatic interactions between the material and EBT molecules [61,76]. The -C=N bond peak experiences a blue shift to 400.9 eV because of π-π interactions between the EBT molecule and the material. An analysis of C1s fine spectra pre- and post-adsorption reveals minimal change in the peak intensity of C-C element. 

However, there is a blue shift in the peaks of the C=N and C-N bonds and a noticeable red shift in the outgoing peak position of the C-OH bond, accompanied by a decrease in the peak area. It can be concluded that the formation of hydrogen bonds, such as O-H·N during EBT adsorption, leads to an increased electron cloud density [78].

**Figure 16:**
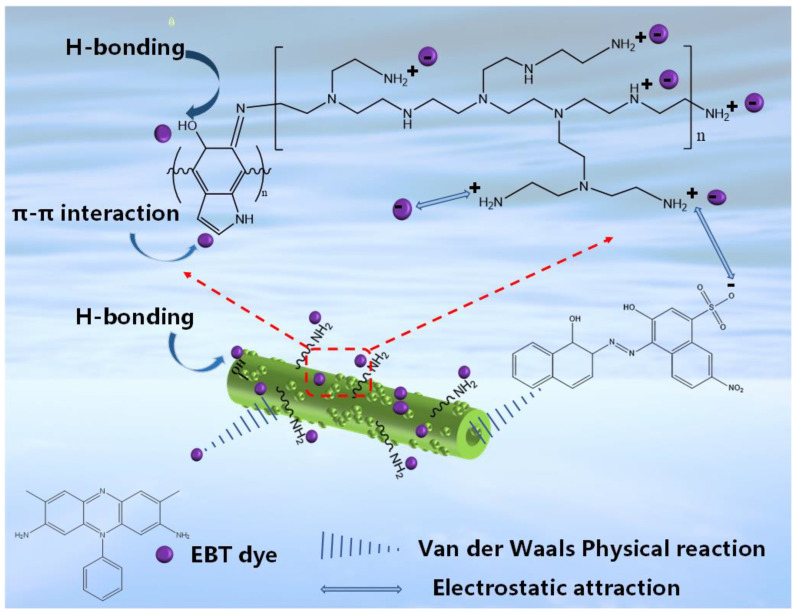
Proposed mechanism of EBT adsorption by PEI-PDA@M-PFs.

In summary, electrostatic interaction, π-π interaction, van der Waals force interaction and hydrogen bonding occurred during the adsorption of EBT dye molecules by PEI-PDA@M-PF, with electrostatic interactions dominating. The simulated mechanism of the adsorption of EBT by the material is shown in Figure 16.

## 4. Conclusions

The polyethyleneimine-grafted mussel-inspired magnetic Platanus officinalis fibers (PEI-PDA@M-PFs) prepared in this paper served as highly effective and recyclable biomass adsorbents for the anionic dye EBT in water. The surface of PEI-PDA@M-PF functionalized with abundant amino groups exhibited a positive charge in aqueous solutions, achieving a theoretical maximum adsorption capacity of 166.11 mg/g at pH 7.0, with an adsorbent dosage of 10 mg. Kinetic and isothermal studies confirmed the adherence and adsorption of EBT on PEI-grafted magnetic fruit fibers, analyzed through pseudo-second-order model, Langmuir model and in-particle diffusion model. Both kinetic and thermodynamic data suggested that adsorption consisted of a chemical and heat-absorbing process forming a monomolecular layer on the PEI-PDA@M-PFs. After multiple cycles of use, the material maintained its exceptional adsorption performance. The adsorption mechanisms revealed that the adsorption process involved van der Waals forces, electrostatic interactions, intermolecular hydrogen bonding and π-π interactions between the samples and EBT dye. The synthesis of PEI-PDA@M-PFs signifies a promising avenue for utilizing natural biomass-derived adsorbents in environmental applications, showcasing considerable potential for practical implementation.

## Figures and Tables

**Table 1:** The composition of chemical elements on the surface of different materials.

Materials	C (%)	O (%)	N (%)	Fe (%)	Co (%)	N/C
M-PFs	60.29	36.46	1.08	1.48	0.69	0.018
PDA@M-PFs	67.89	22.31	5.48	0.62	0.27	0.076
PEI-PDA@M-PFs	57.55	20.75	20.65	0.59	0.44	0.359

**Table 2:** The kinetic parameters and coefficients of the pseudo-first-order model and the pseudo-second-order model for EBT adsorption onto PEI-PDA@M-PFs.

*C*_0_ (mg·L^−1^)	*q*_*e*,*exp*_ (mg·g^−1^)	Pseudo-First-Order Model			Pseudo-Second-Order		
		*K*_1_ (min^−1^)	*q*_*e*,*cal*_ (mg·g^−1^)	*R* ^2^	*K*_1_ × 10^−4^ (g·mg^−1^min^−1^)	*q*_*e*,*cal*_ (mg·g^−1^)	*R* ^2^
50	121.79	0.0096	109.51	0.9572	0.974	124.13	0.9918
75	143.53	0.0150	125.10	0.9156	1.510	137.75	0.9762
100	153.41	0.0161	135.47	0.9385	1.492	148.79	0.9870

**Table 3:** The parameters of the internal diffusion model for EBT adsorption onto PEI-PDA@M-PFs.

*C*_0_ mg·L^−1^	*k*_*d*1_ mg·g^−1^·min^−0.5^	*C*_1_ mg·g^−1^	*R* ^2^	*k*_*d*2_	*C*_2_ mg·g^−1^	*R* ^2^	*k*_*d*3_	*C*_3_ mg·g^−1^	*R* ^2^
50	6.88	2.12	0.9844	2.29	52.62	0.9988	0.67	96.63	0.9134
75	9.91	1.85	0.9896	3.03	59.12	0.9867	1.19	99.79	0.9254
100	11.8	1.48	0.9964	4.29	55.20	0.9665	1.49	100.24	0.9550

**Table 4:** Langmuir and Freundlich isotherm parameters for EBT adsorption on PEI-PDA@M-PFs.

*T*/K	Langmuir			Freundlich		
	*q_m_* (mg·g^−1^)	*b*	*R* ^2^	*K_F_* (L·g^−1^)	*n*	*R* ^2^
298	166.11	0.4204	0.9984	48.65	3.29	0.9456

**Table 5:** Thermodynamic parameters for EBT adsorption onto the PEI-PDA@M-PFs.

	ΔG (kJ·mol^−1^)					ΔH (kJ·mol^−1^)	ΔS (J·mol^−1^·K^−1^)
T	293 K	303 K	313 K	323 K	333 K		
50 mg/L	−2.79	−3.54	−4.23	−4.71	−4.95	13.344	55.54
75 mg/L	−0.21	−0.50	−1.18	−1.47	−1.64	11.114	38.703

## Data Availability

Data are contained within the article.
